# Supplementary material for: Rethinking GPS navigation: creating cognitive maps through auditory clues
Source: Sci Rep. 2021 Apr 8;11:7764. doi: 10.1038/s41598-021-87148-4 (PMC8032695; doi:10.1038/s41598-021-87148-4)
Supplement: Supplementary file 1 — Supplementary Information 1. [file 41598_2021_87148_MOESM1_ESM.docx]

Supplementary Information for

Rethinking GPS Navigation: Creating Cognitive Maps Through Auditory Clues

Gregory D. Clemenson^1,2^, Antonella Maselli^1^, Alex Fiannaca^1^, Amos Miller^1^, Mar Gonzalez-Franco^1^*

^1^Microsoft Research, Redmond, Washington 98052, USA

^2^Department of Neurobiology and Behavior, University of California Irvine, Irvine, California 92697, USA

* Corresponding Author: Mar Gonzalez-Franco

Email: margon@microsoft.com

**This PDF file includes:**

Figures S1 to S7

Legends for Movies S1

**Other supplementary materials for this manuscript include the following:**

Movies S1


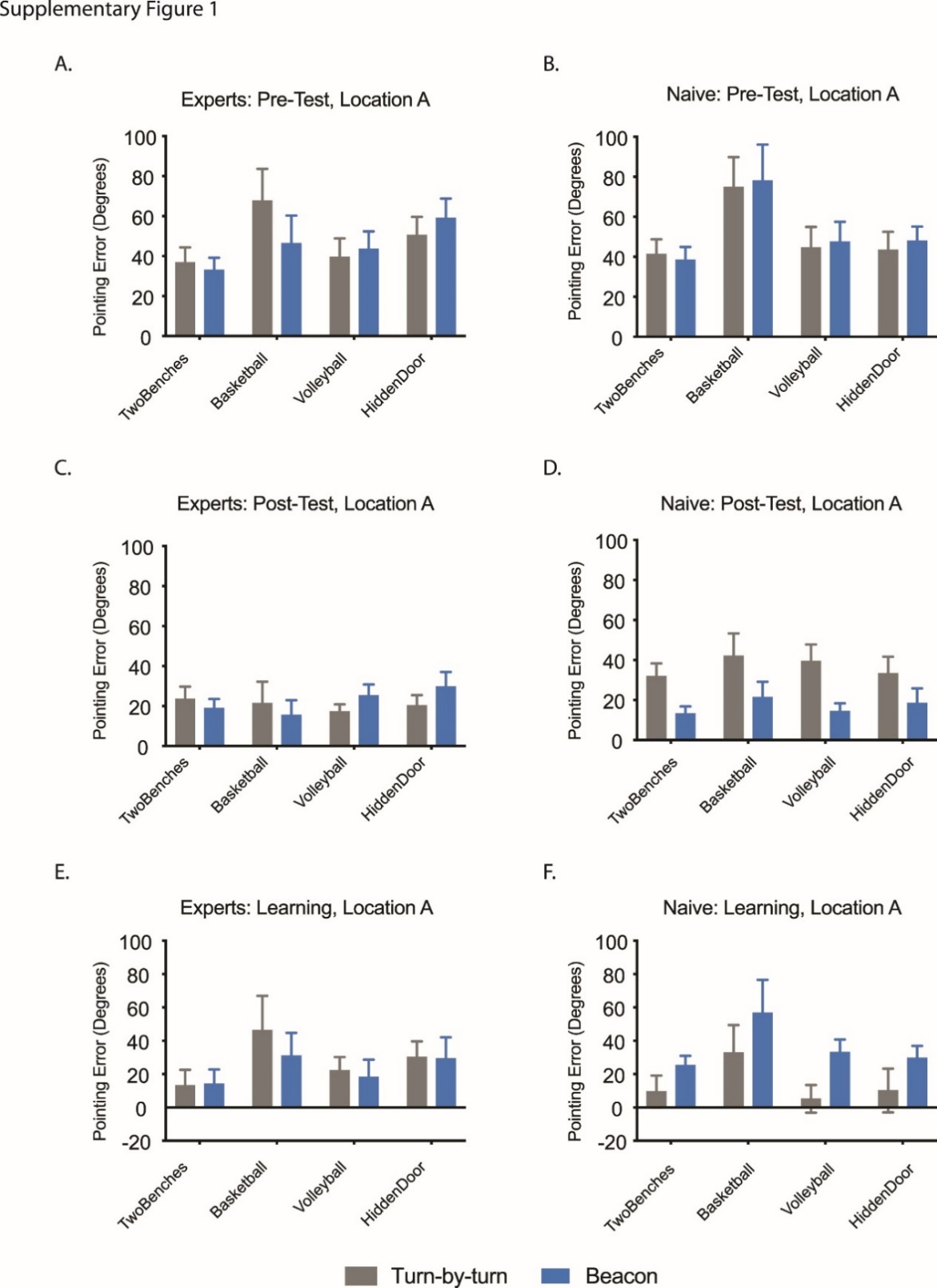


Fig. S1. Individual POI data of both groups from Location A. (A) Pre-Test pointing error of Experts for all individual POIs from Location A. (B) Pre-Test pointing error of Naïve for all individual POIs from Location A. (C) Post-test pointing error of Experts for all individual POIs from Location A. (D) Post-Test pointing error of Naïve for all individual POIs from Location A. (E) The improvement in pointing from Pre-Test to Post-Test of Experts from Location A. (D) The improvement in pointing from Pre-Test to Post-Test of Naïve from Location A.


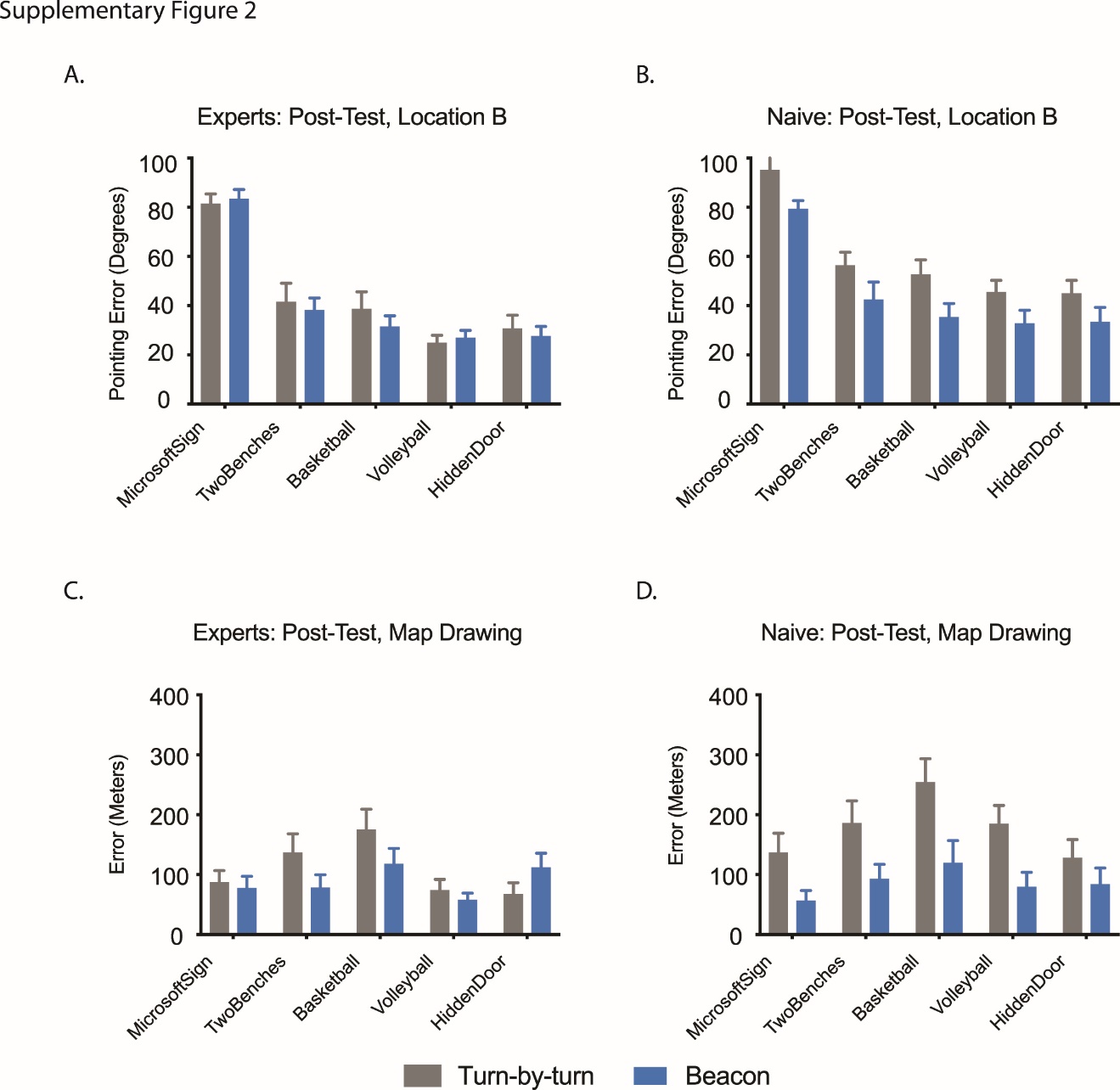


Fig. S2. Individual POI data of both groups from Location B and Map Drawing. (A) Post-Test pointing error of Experts for all individual POIs from Location B. (B) Post-Test pointing error of Naïve for all individual POIs from Location B. (C) Map Drawing performance on all individual POIs for Experts. (D) Map Drawing performance on all individual POIs for Naïve.


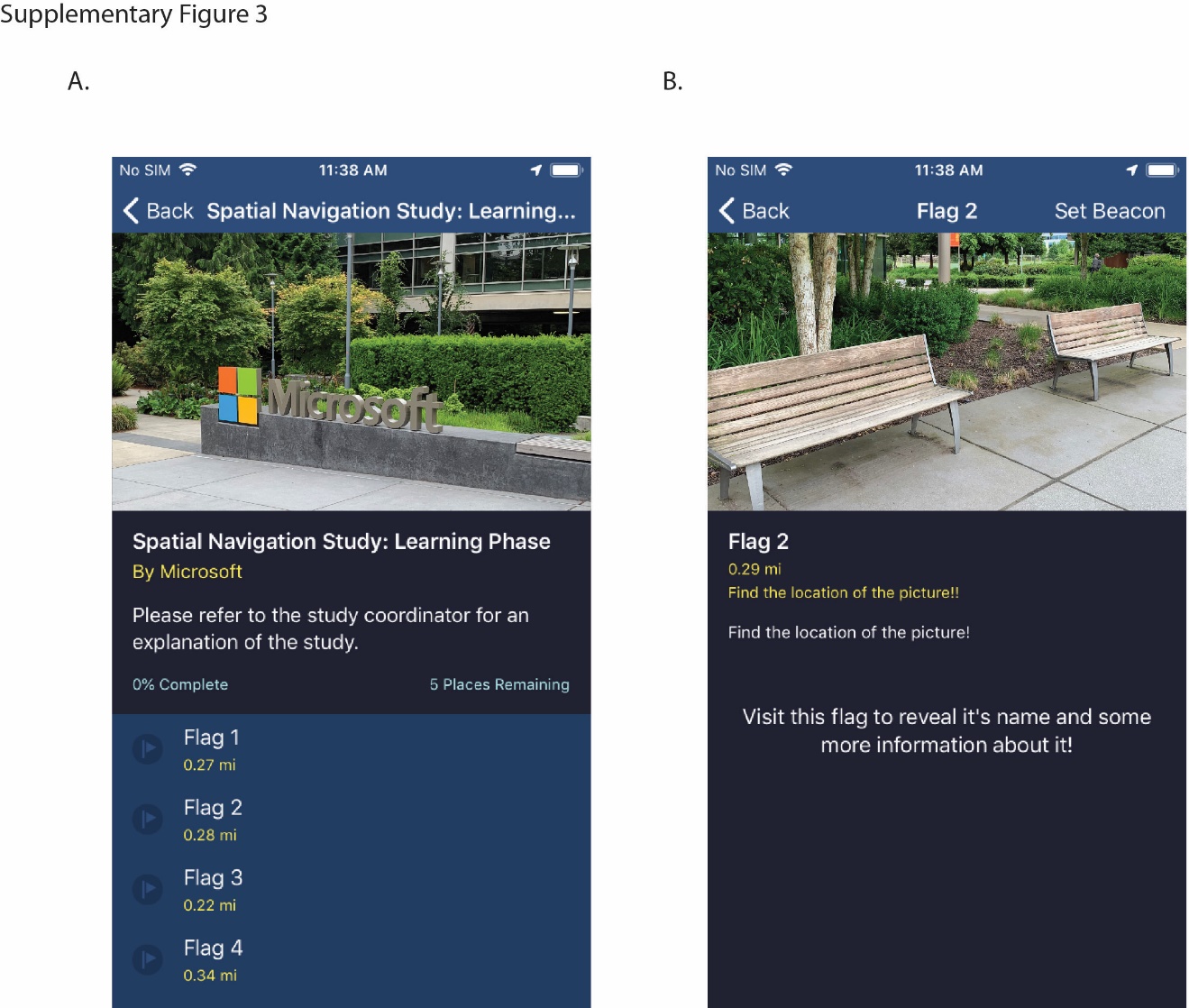


Fig. S3. Example images of the Soundscape app scavenger hunt. (A) Overview of the scavenger hunt and picture of the first POI. (B) After a POI is found, an image of the next one shows up.


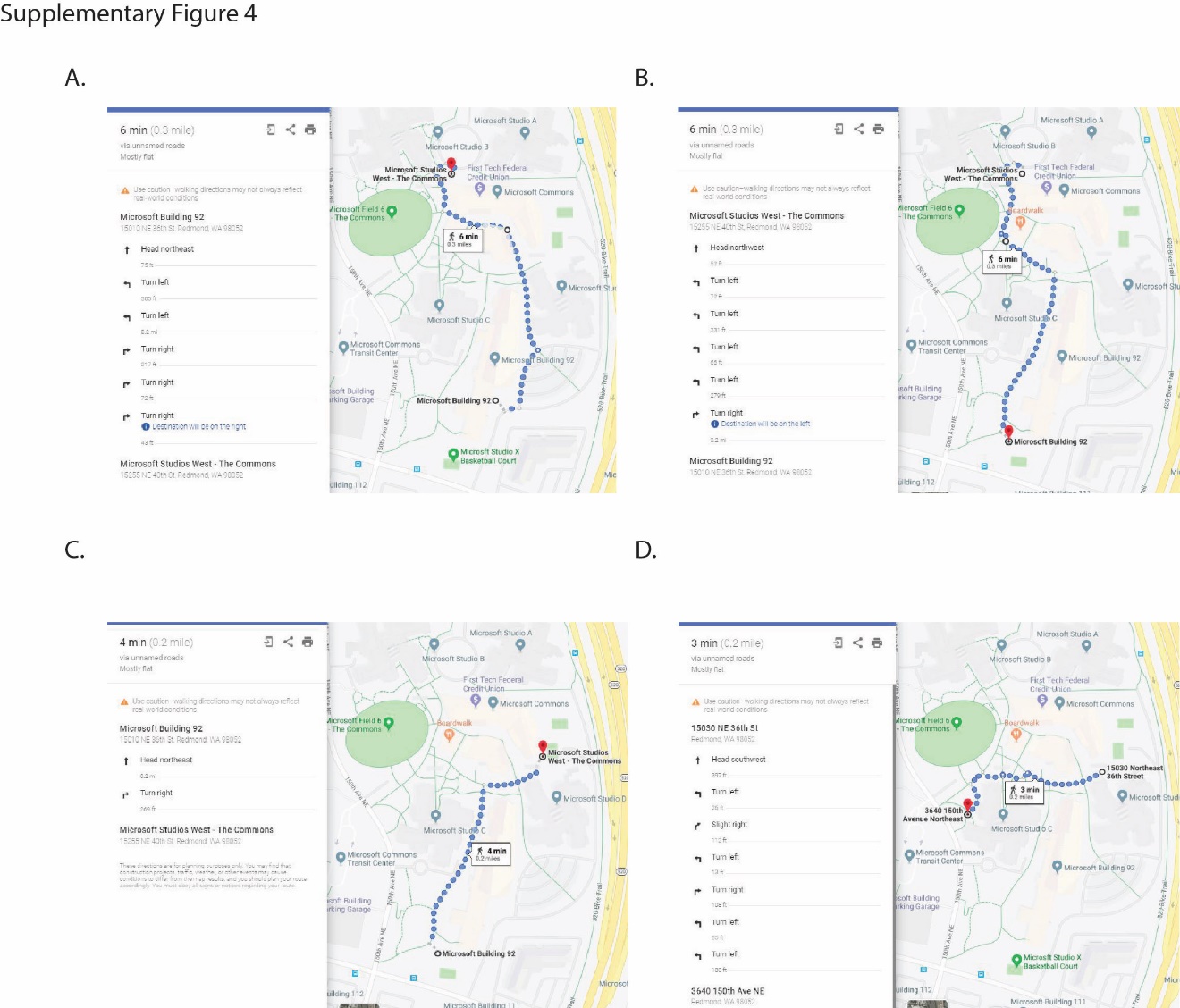


Fig. S4. Directions used for the Turn-by-turn group. (A) Directions from Microsoft Sign to Two Benches. (B) Directions from Two Benches to Basketball Court. (C) Directions from Basketball Court to Volleyball Court. (D) Directions from Volleyball Court to Hidden Door.


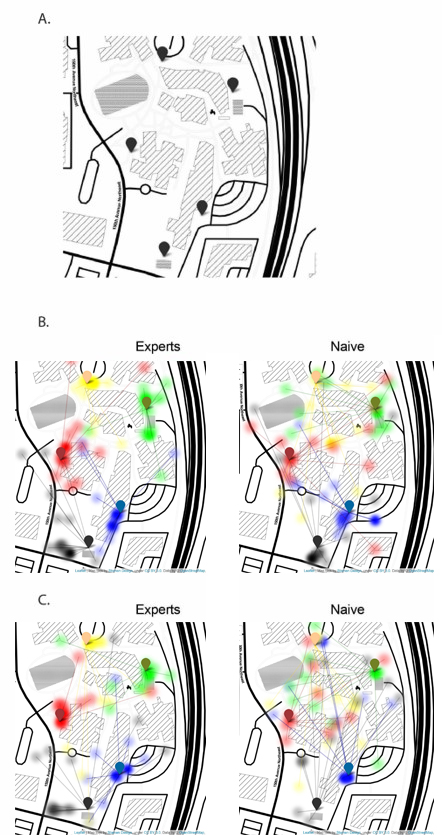


Fig. S5. Map Drawing task and group performance. (A) Simple map with the marked destinations of the scavenger hunt. (B) Performance of both groups in the Beacon group. (C) Performance of both groups in the Turn-by-turn group.


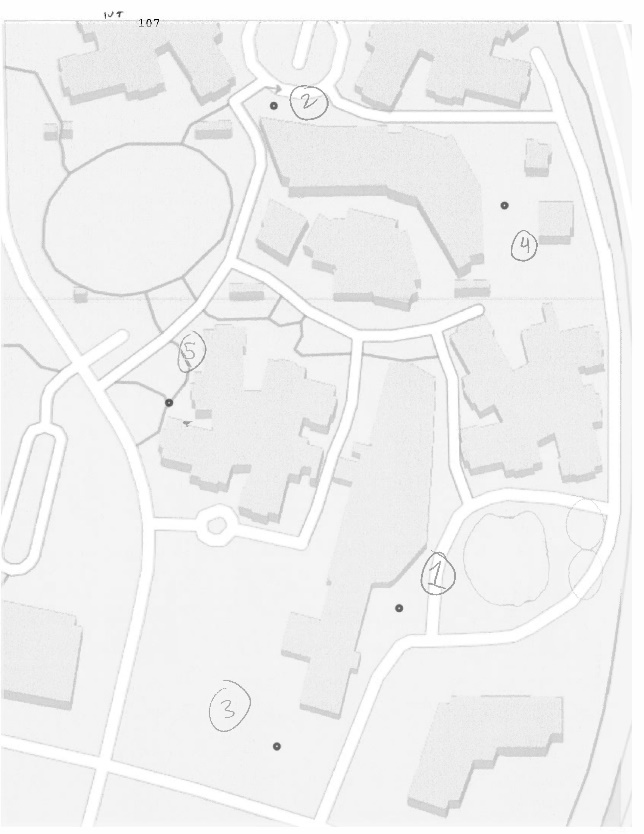

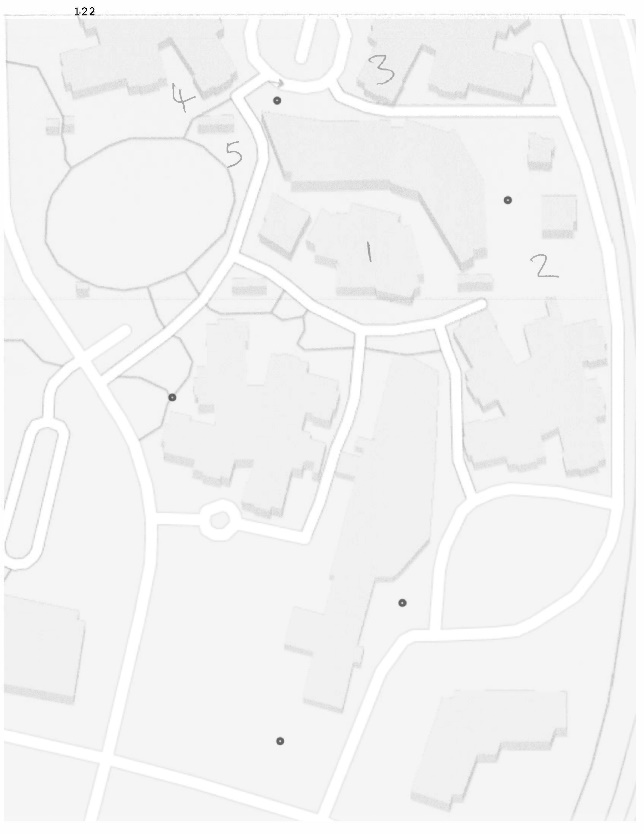


Fig. S6. Real maps with user handwritten numeric locations (numbers of landmarks: 1,2,3,4,5) on the Map Drawing task. For illustration, dots are placed at the scavenger hunt latitude and longitude targets. Left) Drawn map of a participant exhibiting a high-quality mental map, with minimal error between real landmarks and their mental map. Right) The map drawn by a participant who clearly had not developed an accurate mental map after the navigation.


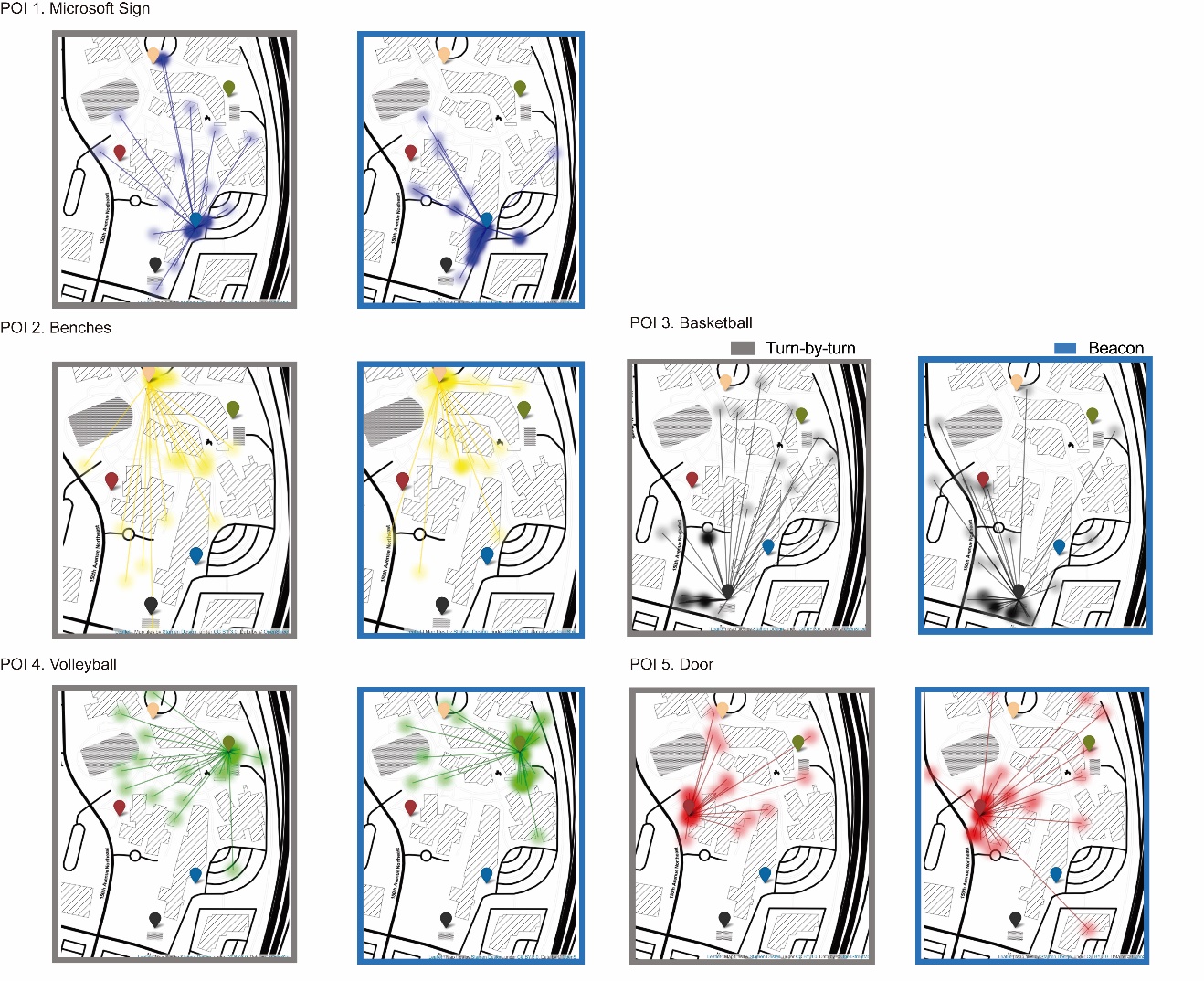


Fig. S7. Map Drawing task and group performance for each POI destinations of the scavenger hunt. For the Turn-by-Turn and Beacon conditions.

Movie S1 (separate file). The video contains an explanation of the results, footage of the participants going through the scavenger hunt and audio of the actual GPS app.
